# Quantifying Phenotypic Variation in Isogenic *Caenorhabditis elegans* Expressing *Phsp-16.2::gfp* by Clustering 2D Expression Patterns

**DOI:** 10.1371/journal.pone.0011426

**Published:** 2010-07-19

**Authors:** Alexander K. Seewald, James Cypser, Alexander Mendenhall, Thomas Johnson

**Affiliations:** 1 Seewald Solutions, Vienna, Austria; 2 Institute for Behavioral Genetics, University of Colorado at Boulder, Boulder, Colorado, United States of America; Centre for Genomic Regulation (CRG), Universitat Pompeu Fabra, Spain

## Abstract

Isogenic populations of animals still show a surprisingly large amount of phenotypic variation between individuals. Using a GFP reporter that has been shown to predict longevity and resistance to stress in isogenic populations of the nematode *Caenorhabditis elegans*, we examined residual variation in expression of this GFP reporter. We found that when we separated the populations into brightest 3% and dimmest 3% we also saw variation in relative expression patterns that distinguished the bright and dim worms. Using a novel image processing method which is capable of directly analyzing worm images, we found that bright worms (after normalization to remove variation between bright and dim worms) had expression patterns that correlated with other bright worms but that dim worms fell into two distinct expression patterns. We have analysed a small set of worms with confocal microscopy to validate these findings, and found that the activity loci in these clusters are caused by extremely bright intestine cells. We also found that the vast majority of the fluorescent signal for all worms came from intestinal cells as well, which may indicate that the activity of intestinal cells is responsible for the observed patterns. Phenotypic variation in *C. elegans* is still not well understood but our proposed novel method to analyze complex expression patterns offers a way to enable a better understanding.

## Introduction

In Rea et al. [1], we have found that average activity of *hsp-16.2* correlates well with lifespan in adult worms. More precisely, within isogenic populations of adult *C. elegans* expressing *Phsp-16.2::gfp* and having developed in the same environment, worms with higher average GFP intensity after heat shock tend to live significantly longer than those with a lower average GFP intensity. There remains a suprisingly large amount of phenotypic variation in the expression of this protein which merits further study.

However, the Copas Biosort worm sorter which we used for these experiments is unable to resolve intensity variation along the lateral axis. Only intensity variation along the antero-posterior axis could potentially be measured. Dupuy et al. [2] show just such an analysis for *C. elegans* promotor activity from early larvae to adult. As we wanted to study the intensity variation along both axes, we had to develop a different approach. The main idea was to use worm microscopy images to obtain intensity variation images automatically by developing appropriate image processing methods.

As a first step, we prepared an adult worm population as in Rea et al. [1]. We took both DIC and GFP intensity microscopy images of individuals after anaesthetizing them and placing each on a separate clean slide. In most cases, we took one image of the anterior and one of the posterior part of each animal, with some overlap (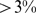) between these two images. We developed image processing algorithms to combine these two images, to determine pixels within and outside of the worm body, and to extract a two-dimensional intensity image for each worm by overlaying a grid over the worm body. These intensity images were uniformly arranged along both axes (top: anterior, bottom: posterior, left: left lateral, right: right lateral/vulva) so that they could be compared between different worms.

The same method could also be used to enhance weak GFP intensity patterns by merging intensity images over a large number of worms; quantify phenotypic variation for other GFP reporters using a similar hierarchical clustering approach; quantify GFP reporter variation of genetically different strains while distinguishing between phenotypic and genotypic variation; quantify activity of different reporters by comparing the averaged and/or clustered intensity images and so on.

Initially we tested the worm straightening algorithm used in Peng et al. [3]. However, their system does not normalize worm width and thus the two-dimensional intensity image would have pixels without any intensity values which severely complicates clustering. Additionally when applying their approach to our images, the estimation of the worm “backbone” failed or was incorrect both for the known binary worm image (as output by our algorithm) as well as for the raw GFP images in the majority of cases. The reason for this might be that they mainly focus on nuclear GFP reporters which have point-like responses while our reporter is active at varying intensity levels throughout the entire cytoplasm of most cells.

Guberman et al. [4] describe another system which offers similar functions for single bacterial cells. While their internal coordinate estimation approach has a quite similar goal as our two-dimensional intensity images, their approach to find contours is not suitable for *C. elegans* because – contrary to bacterial cells – worms are not of uniform brightness when imaged via phase contrast microscopy but show significant variations in brightness within the worm body. These variations yield many false contour points within the worm body which prevented us from applying their approach as-is.

Our main aim was to analyze phenotypic variation by image processing of microscopy images of animals expressing *Phsp-16.2::gfp* in a manner that is independent of average activity. We found clusters that were consistent with previous results based on average activity measurements from Rea et al. [1], but showed a more complex structure, with the bright worms being assigned to one cluster and the dim worms being separated into two clusters with distinct expression patterns.

Based on a preliminary confocal analysis of five bright and five dim worms, we found that these activity loci were caused by extremely bright intestinal cells. We also found that the vast majority of signal from the *Phsp-16.2::gfp* reporter transgene originated in the intestine cells, suggesting that the observed patterns are caused by intestinal cells. We speculate that the high average intensity signal of long-living worms may also be caused by these small cell clusters that we could trace to specific cells (see Discussion).

As a secondary aim, we were interested in comparing our method to other approaches. We have therefore compared a simplified use of our method to average intensity results from a Copas Biosort worm sorter and found very good agreement. This is a minor result which nevertheless lends some additional support to our new method.

## Methods

This section describes the image processing algorithms to determine pixels corresponding to the worm via a trained classification model operating on image pixels (*pixelClassification*); mesh (i.e. combine with overlap) anterior and posterior worm images (*meshAB*); and extracting axis-aligned worm intensity images from non-normalized image data (*sampleCE*). All algorithms are available on http://elegans.seewald.at. The preparation of the worms was done exactly as in Rea et al. [1] and is described there.

### Terms

An image 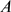 is represented by the two-dimensional matrix 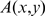, where x is the zero-based column index, y is the zero-based row index and the value of 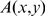 corresponds to the pixel value at the position 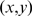.

The Normalized Correlation Coefficient (NCC) over a template 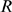 and an image 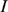 at position 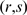 (i.e. the template 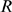 is laid over the image 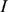 such that the top-left corner 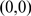 of 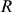 corresponding to 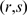 of 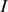) is defined as follows. NCC is equivalent to a Pearson's correlation coefficient on the same data assuming the full population formula, because in that case 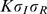 is the denominator and simplifies to the formula below.
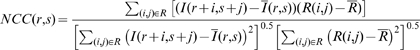
(1)

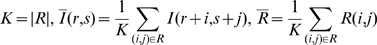



### pixelClassification

Input images were first equalized by histogram equalization (see Burger et al. [5]), obtaining a flat histogram. This operation vastly improves the constrast of dark images. Then we trained a logistic regression function (see le Cessie et al. [6]) to recognize worm pixels. We initially used eight GFP sample images, manually determined worm pixels for each image, and computed average and relative standard deviation of pixel values in a 5×5 window around each pixel. This data was used to build our first model, which was then subsequently refined. The used features were inspired by Geng et al. [7] who used similar features to recognize worm pixels, albeit in low-resolution DIC images. In initial experiments, GFP images performed much better as base images for this detection than DIC images. The reason for this is most likely the autoflourescence of the worm itself which can be readily distinguished from the background.

Our trained logistic regression model outputs the probability of a worm pixel at each image position 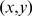. Initially, we used a fixed threshold to determine whether a pixel was to be considered part of the worm (i.e. if the model returned a value greater or equal to the threshold, the corresponding pixel was considered to be part of the worm). However, this soon proved to be too inflexible for the wide variety of images which we processed.

Therefore, we chose to test a large set of empirically chosen thresholds to determine whether the resulting shape was sufficiently worm-like. To determine worm-likeness of the final shape, we first removed all shapes but the largest 8-connected shape, yielding one candidate shape per threshold. Among all candidate shapes determined via different thresholds, we considered the shape with at least 3% and at most 25% area (measured as proportion of total image pixels) and with the highest circularity (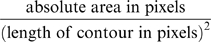, see Burger et al. [5]) as the best worm-like shape.

Finally, all inner contours within the final shape were filled. The shape was eroded three times, then dilated three times (erosion and dilation see Burger et al. [5]) to remove small border irregularities and afterwards larger irregularities in the contour were filled by computing local contour tangents and correcting parts of the contour which deviate too much from local contour curvature. Large irregularities were almost always eggs clinging to the worm body.

### meshAB

The combination of anterior (A) and posterior (P) worm images with overlap relies on knowing the set of pixels belonging to the worm body. Therefore it needs images already processed by *pixelClassification* as input. These images have both normalized 8bit GFP intensity data in the green channel, and worm pixel membership data in the blue channel for three classes: worm border (one-pixel thin, 8-neighborhood), worm interior, and worm exterior (pixels outside the worm, background).

First, both A and P images are each cut down to the smallest rectangle which contains all worm pixels. This reduces the computational effort for the next steps.

It is assumed that both images were taken at the same resolution. Therefore, combining both images can be done with a simple model containing two shift values: one for the x axis and one for the y axis. The following computation relies only on the green channel of the image. The best shift values are computed by a two-level hierarchical search using NCC (see Formula (1)).

First, all shift values which are a multiple of 16 are tested. In the second step, all integer shift values in an interval of 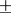
16 pixels around the previous best shift values are tested to determine the final best shift values 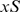 and 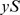. We also tested estimating shift values and combining at subpixel resolution but results were not significantly better.

Next, we estimate a linear regression function to remove brightness changes between both images. Note that this is only done for improving segmentation results and that the final intensity image is computed from non-normalized data. We estimate parameters 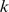 and 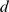 from all pixels within the overlapped area via least-squares fitting of 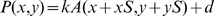 (i.e. selecting 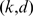 to minimize 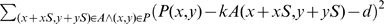.

The final combined image C can then be computed as follows. Since we have two pixel values in the overlapped areas, these need to be averaged via 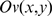.
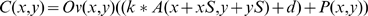
(2)

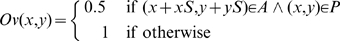



The process thus far only computes the green channel of the resulting combined image. To also compute the new blue channel containing worm membership, we first remove the worm border from both source images and afterwards combine worm interior pixels from both images as follows: a worm interior pixel in either 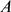 or 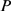 yields a worm interior pixel in the combined image (i.e. combination using logical OR).

By morphological “skeletonization” via thinning (i.e. reducing the worm body to the one pixel thin worm “backbone” by repeated erosion, see Burger et al. [5]) of the now binary blue channel we can obtain the two endpoints of the worm, one of which must be head and the other must be tail. However, since the combination might yield border irregularities, more than two endpoints may be found. We removed ambiguity from the skeleton using the approach described in Geng et al. [7] by first shrinking the “skeleton” from all endpoints simultaneously until only two endpoints exist, and then extending these to endpoints towards the border (i.e. reversing their previous shrinkage). In most cases these can be unambiguously assigned to head and tail endpoints (except e.g. for curled up worms) by using the known assignment of source images to anterior (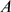) and posterior (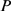) of the worm.

To determine the lateral side for the vulva depending on worm pose, we considered the following two rules:

- Given a convex worm “backbone”, the vulva should always be on the inner side (i.e. the side that is more strongly curved) of the convex worm.
- The vulva should always be on the left side w.r.t. the worm head (i.e. always left lateral)

We tested both rules on sample images and found that the first one is always correct while the second one is only correct in 70% of cases. In the erroneuous cases the worm was upside down, which likely happened when moving the worm to a clean slide. So we implemented only the first rule here which should give a reliable lateral side estimate for the vulva about half of the time. In the remaining half, the worm “backbone” is not convex. Note that an exact position of the vulva cannot be obtained currently because image resolution of our samples is insufficient to automatically recognize anatomical features. This is therefore left for future work.

### sampleCE

Based on final tagged images either from *pixelClassification* or from *meshAB* (via *pixelClassification*) and original non-normalized GFP intensity images, this algorithm computes two-dimensional intensity images.

First, the skeleton and the skeleton endpoints are obtained using the same approach as in *meshAB*. The endpoints are assigned to the head and tail using the known head and tail positions (either obtained via *meshAB* or determined manually). The vulva position is also known at this point and if necessary the lateral axis is mirrored to ensure a consistent positioning of the vulva to the right of the image.

**Figure 1:**
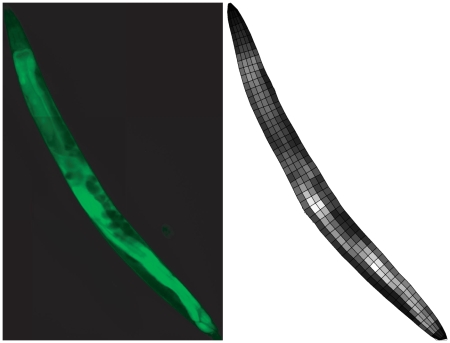
Demonstration images showing quadrangle grid decomposition by the *sampleCE* algorithm. Left image shows meshed GFP intensity base image (created with *meshAB* from two separate images). Right image shows the worm overlaid with a grid of quadrangles. Each quadrangle is filled with the average intensity value computed from the corresponding parts of the base image and determines one pixel in the final 2D intensity image (width = 5, height = 75 was chosen for here; white = maximum intensity, black = minimum intensity). Quadrangle borders are shown in black for clarity.

Afterwards, the skeleton is tracked from head to tail in steps corresponding to the desired height of the 2D intensity image. At each point, the local worm “backbone” tangent is computed and the border of the worm body is searched in both directions perpendicular to this tangent. The space between both worm borders is split into a number of equally spaced intervals corresponding to the desired width of the 2D intensity image. This process results in a set of non-overlapping quadrangles which cover the entire worm body. Each quadrangle corresponds to one pixel of the resulting 2D intensity image. The pixel values are computed by averaging over the GFP intensity values within each quadrangle. Fig. 1 shows a sample. The intensity image is created by collecting all averaged GFP intensity values in the order determined by the previous alignment of anterior-posterior and left lateral/right lateral axes.

## Results

We first describe the preparation of 2D intensity images, and then proceed to describe how we clustered these 2D intensity images. For a second minor result, we also collected a second set of worms, again computed 2D intensity images but then averaged each image to a single value, and compared the results to the output from a Copas Biosort worm sorter.

### Preparing 2D intensity images

For the following analysis, we took an isogenic population of *C. elegans* specimens of strain TJ375 which express *Phsp-16.2::gfp* and followed the protocol for preparing them for microscopy (see Rea et al. [1]). The specimens were sorted into three sets: *Bright* (B, 19 specimens), a random sample from the 3% brightest specimens; *Dim* (D, 24 specimens), a random sample from the 3% dimmest ones; and *All* (A, 25 specimens), a random sample from the remaining worms equally distributed over the full range of observed brightness values excluding top 3% and bottom 3%. Sorting of the worms was performed with a Copas Biosort worm sorter. If possible, the microscopy image was taken with the full worm in one image, otherwise separate anterior and posterior worm images were taken and later combined via *meshAB*.

We had to remove three images from *Bright*, two images from *Dim* and three images from *All* for the following reasons.

- B02 was taken as anterior and posterior image, and there was a difference of factor 5 between image intensities in the overlap between both images. This indicates photobleaching or different exposure settings between the images and makes absolute intensity values suspect.
- B17, D24 were missing the tail in an image supposedly containing both parts of the worm.
- B01 was an image of the same worm as B05, clearly visible as the largest peak in pairwise worm correlation, so we did not include it in the analysis. However, we used this image to compute the best resolution for the 2D intensity images (see below).
- D17 was removed because as only image it did not fit the intensity ordering from *Bright* to *Dim* and thus was considered an outlier. Different exposure settings may also have been responsible.
- A02 was too dark, so the agreement of worm position between GFP and DIC image was unclear. To prevent analyzing parts of the background instead of the worm specimen, we removed this image.
- A10, A17 had unclear vulva position even after expert check. Two experts disagreed on vulva position as the DIC image was too blurry and the vulva did not show clearly in the GFP channel. This would have prevented unambiguous lateral axis determination and therefore these images also had to be removed.

Some of these reasons were due to human error and could be prevented in the future by optimizing image acquisition (see Discussion).

Images were analyzed by our automated system. All images were first processed by *pixelClassification* followed by minor manual corrections of worm shape in about one sixth of the cases. Head and tail position were automatically determined via *meshAB* where head and tail images were available. In 40% of these cases, vulva position could also be obtained automatically. For the remainder, head, tail and vulva position were manually determined. All images were additionally checked by two of the authors.

We chose to sample a 75 pixels (for the anterior/posterior axis, i.e. height) by 15 pixels (for the left lateral/right lateral axis, i.e. width) intensity image via *sampleCE*. This resolution yielded the best trade-off between high activity pattern resolution and low divergence of anatomical features between worms. Tests to determine this resolution were done by analyzing two images of the same worm (B01,B05) and detecting at which resolution the correlation (measured via NCC) between the two intensity images dropped significantly. This drop indicates a misalignment of fine anatomical features between both images, so the highest resolution before this drop (width = 15, height = 75 pixels) was used to obtain best alignment between different worms. The aspect ratio of 1∶5 was chosen to roughly correspond to the real aspect ratio of the worm with a slight bias towards higher resolution along the left/right lateral axis.

### Clustering 2D Expression Patterns

To determine similarities between our worm specimens based on 2D intensity patterns, we chose to use a hierarchical clustering algorithm, *hclust*. For bootstrap sampling confidence measures to determine the stability of the clustering, we used *pvclust* which uses *hclust* internally. *pvclust* computes approximately unbiased probability values for each cluster by multistep-multiscale bootstrap resampling (see Simodaira [8], [9], Suzuki et al. [10]). We used the free implementation of these clustering algorithms from the R project [11].

Since the *Bright* and *Dim* images were already known to differ in average intensity, we chose a distance measure which is independent of average intensity: NCC (see formula (1)). We used 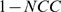 as distance measure for hierarchical clustering. NCC is equivalent to the well-known Pearson's correlation coefficient under weak assumptions. Our clustering distance measure abstracts from relative changes in intensity and is thus relatively robust to changes in autofluorescence between worms.

When clustering with 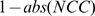, the clusters remained exactly the same. When clustering with euclidean distance as the measure, we obtained three different clusters: one with all *Bright* images except B11 plus A23, A24; one with all *All* images except A21, A23–A25 and one with all *Dim* images plus A21, A25, B11. As euclidean distance is not independent of average intensity, this clustering almost perfectly reconstructs the original selection of *Bright*, *Dim* and *All* by average intensity and does not yield significant information beyond that.

**Figure 2:**
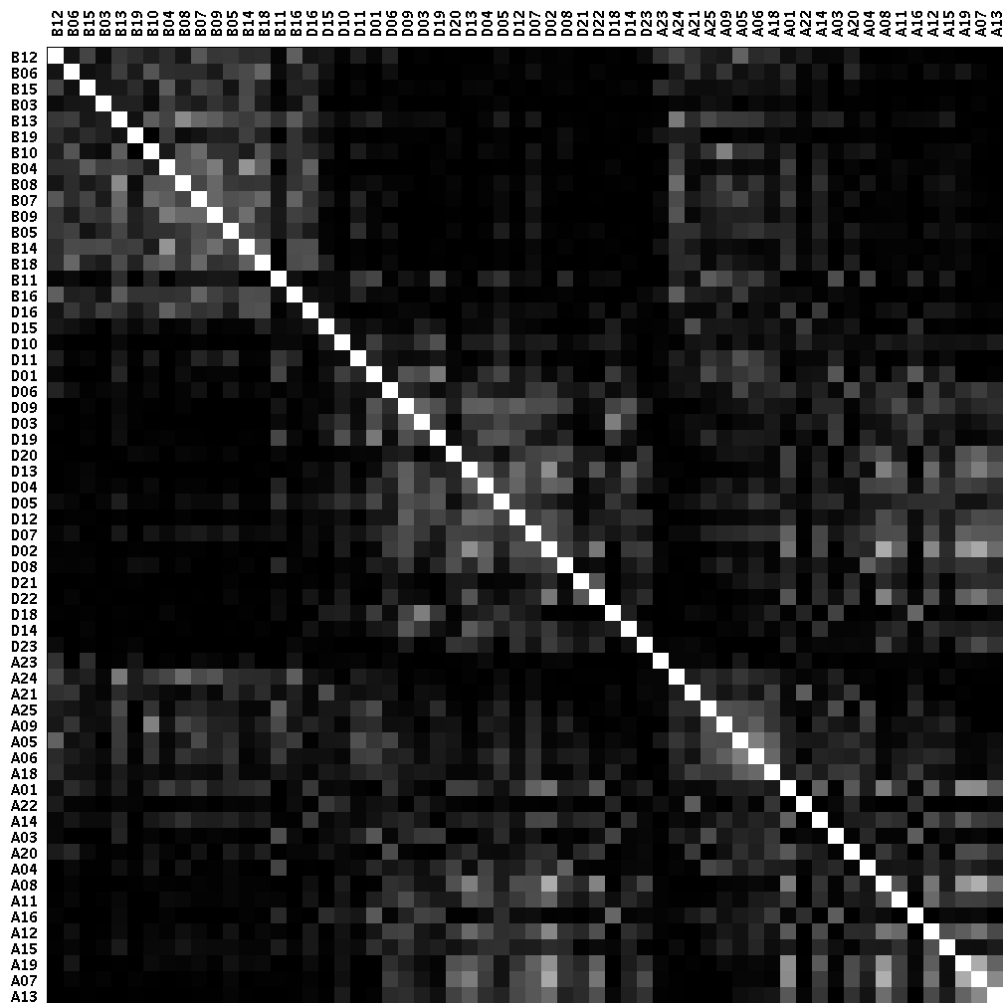
Correlation matrix of all worms. Row and column labels show worm names (*A = All*, *B = Bright*, *D = Dim*) and are sorted by label (*B,D,A*), and then by average squared NCC (summed over row/column) descending. Each cell shows correlation with white = maximum correlation = 1.0, black = minimum correlation = 0.0, computed via squared NCC. One cluster for *B* worms and one cluster for *D* worms can be easily discerned. It can also be seen that *A* worms don't show clear clusters as a group. There are however some *B*-like patterns at the top and some *D*-like patterns at the bottom of the *A* group.

Fig. 2 shows a correlation matrix of our set, which shows the three groups *B(right)*, *D(im)* and *A(ll)*, computed via NCC. Within each group, specimens were sorted by descending average NCC (summed over rows/columns) to clarify the pattern. The *Bright* worms and the *Dim* worms showed distinct clusters which correlate within each group but not between the two groups. For the *All* group, we also found some *Bright*-like patterns on top and some *Dim*-like patterns at the bottom - and also an area in between where neither *Bright* nor *Dim* patterns are well correlated, as would be expected. It seems that the distinct patterns for *Bright* and *Dim* worms are valid somewhat beyond the 3% highest/lowest intensity specimens.

**Figure 3:**
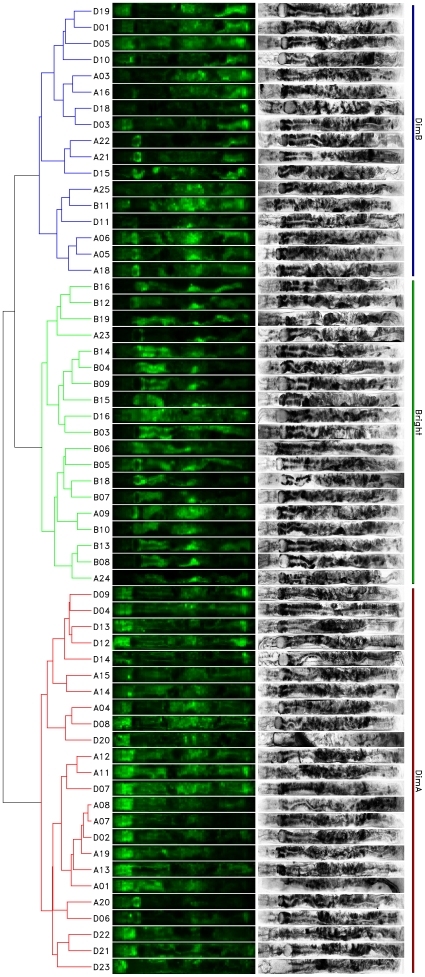
Hierarchical clustering of all worms (A = *All*, B = *Bright*, D = *Dim*). Left shows hierarchical clustering with two clusters for *Dim* (*DimB* = blue with confidence 65%; *DimA* = red, 70%) and one cluster for *Bright* (*Bright* = yellow, 61%). Second column shows GFP 2D intensity images with w = 15, h = 75; third column shows DIC 2D intensity images with w = 52, h = 520 (rotated 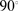 to the left). Fourth column shows cluster names and extents for clarity. Each row is a distinct specimen from our experiments. Note how the DIC images allow the recognition of gross anatomical features. All images are aligned with head to the left, tail to the right and vulva to the top. The set of clusters with at least 95% confidence is D06+A20, A07+A08, B08+B13, B10+A09, B04+B14, A18+A05+A06, B11+A25, D15+A21+A22, D03+D18, A16+A03 and D01+D19, however these are too small for a systematic analysis.

To enable a more detailed analysis, we computed the hierarchical clustering shown in Fig. 3. In addition to the specimen ids, we also show the complete GFP 2D intensity image (sampled at 75×15 pixels), and also the DIC 2D intensity images for each worm (sampled at 520×52 pixels). Both were normalized per specimen. Although there was only one cluster for *Bright*, there were two distinct clusters for *Dim*: *DimA* and *DimB*. The *All* worms were distributed among all three clusters. These three clusters had confidence estimates of *DimA* = 70%, *DimB* = 65% and *Bright* = 61%. When we considered only those clusters with at least 95% confidence, we obtained nine clusters of two specimens each and three clusters of three specimens each. These were D06+A20, A07+A08, B08+B13, B10+A09, B04+B14, A18+A05+A06, B11+A25, D15+A21+A22, D03+D18, A16+A03 and D01+D19. However, because of their small size these could not be systematically analyzed.

As a large part of the *All* worms could not be reliably assigned to either *Bright* or *Dim* clusters (noted earlier), we assumed that the random assignment of unclear *All* specimens to either of these clusters was responsible for the low confidence estimates. This was confirmed by clustering only *Dim* and *Bright* worms, where we obtained exactly the same clusters (except for the missing *All* specimens) with *DimA* = 84%, *DimB* = 90%, *Bright* = 88% – a drastic increase in confidence. All clusters with at least 95% confidence from the previous clustering that did not contain an *All* specimen were also present and all of them had 100% confidence. So we may also conclude that worms with intermediate brightness levels do not display patterns as stable those specimens at the extremes.

**Figure 4:**
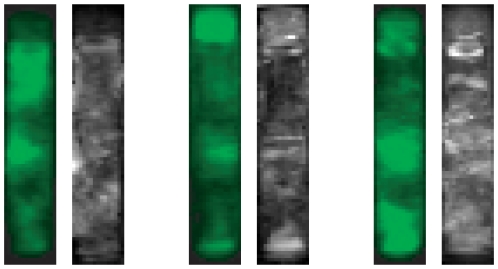
Average worm activity of clusters *Bright*, *DimA* and *DimB* (top = head, bottom = tail, vulva to the right). Each group shows average GFP intensity (left) and relative standard deviation of avg. activity (right). Left images: *Bright* has a spot near the middle of the worm on the other side of the vulva; *DimA* has a spot near the head and *DimB* has a spot near the tail and near the center (less intense than *Bright*). Right images: the peaks mostly correspond to slight misalignments of anatomical features, e.g. pharnyx in *DimB*. The absolute values of standard deviations is quite small, but the images were normalized to enhance the visible patterns. We have taken extra care to recheck all axes.

In order to better characterize these three clusters, we have averaged the GFP activity over each group. Fig. 4 shows the result. Distinct activity loci could be observed (left in each group), especially one locus for *Bright* at the vertical position of the vulva, but on the other horizontal position (i.e. opposite the vulva), and one locus near the pharynx. *DimA* had stronger activity towards the head while *DimB* had stronger activity towards the tail and to a lesser extent in the abdomen. This can also be seen by referring to the original images in Fig. 3, where most worms of each cluster exhibit these cluster-specific patterns.

The standard deviation of average activity (shown right in each group) shows different aspects. The areas with high standard deviation point out areas where the alignment of anatomical features is less accurate – the pharynx in *DimB*, the area near the tail in *DimA* and the area opposite the vulva in *Bright*. The last may be an artefact of the high peak in average activity near this position.

Concluding, we have noted three distinct clusters for the expression of *Phsp-16.2::gfp*, one of which was correlated with high average intensity worms and two of which were correlated with low average intensity worms. Note that an 1D analysis using a worm sorter would not have allowed us to determine that the exact position of the activity locus near the vulva in the *Bright* cluster was not at the vulva but on the left lateral side opposite the vulva.

To follow up on our finding, we conducted a preliminary confocal analysis of five bright and five dim worms, and found that the vast majority of signal from the *Phsp-16.2::gfp* reporter transgene originated from intestine cells, including the major activity loci from the three clusters we found. We speculate that these activity loci may be caused by intenstinal cells, and these cells may also be responsible for the high average activity of the *Bright* worms (data not shown, more details in Discussion).

### Validation vs. Worm Sorter

To validate our method, we randomly selected a second set of 23 worms (prepared as in Rea et al. [1]) and ran them through a worm sorter before putting them on a slide and capturing microscopy images in the same way as described above. Again, we recorded both DIC and GFP images. Note that this analysis reduces the 2D intensity image created by our method to a single value.

In nine cases, the whole worm was captured in a single image. The remaining worms each had an anterior and posterior image. We checked the anterior/posterior images' average GFP activity in the overlapping area of each worm and removed those five worms for which this difference was more than 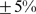 (the acceptable deviation here is much smaller than for the previous experiment as the following analysis does make use of absolute intensity values). Presumably these differences were caused by different settings (e.g. camera exposure, focus or GFP excitation intensity).

For the remaining 18 worms, we again automatically merged the anterior/posterior images in order to obtain one image per worm via *meshAB*. This image was then analyzed by *sampleCE* to determine 2D intensity images. We computed the average GFP activity by worm as the average of pixel values over the full intensity image (width = 15, height = 75 pixels as before). This was done to get values which could be compared to the worm sorter.

**Figure 5:**
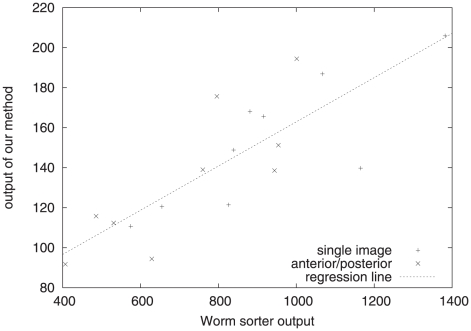
Scatter plot of avg. GFP activity. The x axis shows worm sorter measurements, the y axis shows measurements due to our method (one average intensity value averaged from each 2D intensity image). Each data point is a sample, + marks those worms which were captured in a single image, x marks those which were combined via *meshAB* from two separate anterior/posterior images. The shown regression line indicates good agreement (r = 0.8167). While the same data could be obtained more efficiently using the worm sorter, it gives additional support to our method.

Fig. 5 shows good agreement between both measurements. The correlation coefficient is 0.8167. There was no difference in the correlation of merged (anterior/posterior) and non-merged worm images so *meshAB* does not impair the measurement of average GFP intensity in the merged images.

The signal-to-noise ratio could be improved by increasing GFP excitation intensity or exposure time. Note that although a camera with 12 bit resolution was used, the highest pixel value we observed was only a third of the possible maximum value.

Of course our method extracts far more information than the single value obtained via a worm sorter. However, the good agreement of our reconstructed average intensity values lends additional support to our method and also demonstrates that two different ways to measure the same intensity give comparable results.

## Discussion

Herein we have presented our efforts to develop a method for extracting additional data from fluorescent micrographs of the nematode worm, Caenorhabditis elegans. This is especially timely since we have discovered that expression from a transgenic strain carrying an integrated reporter (*Phsp-16.2::gfp*) can be used to predict subsequent longevity and stress resistance in Rea et al. [1]. We have shown that using digitized information we can observe patterns of *hsp-16.2* expression that distinguish the *Bright* and *Dim* worms that are destined to have differential longevity. These differential clusters were seen after we normalized the levels of expression between *Bright* and *Dim* and were validated in several ways.

We find that *Bright* and *Dim* worms are not only different quantitatively but that qualitative differences in the cells that express *hsp-16.2* seem to also underlie the quantitative differences in expression that predict subsequent longevity. The highest levels of expression of the reporter were in the intestinal cells and our preliminary evidence suggests that there are specific sets of intestinal cells that tend to be hotspots of expression in the *Bright* cluster, *DimA* cluster, and *DimB* cluster. In the *Bright* animals, the most *Phsp-16.2::gfp* expression emanated from cells comprising the first intestinal ring (cells intDL, intDR, intVL, and intVR) and the fifth intestinal ring (int5L and int5R); the same pattern is apparent in *DimA* clustered animals. However, the expression pattern of *DimB* animals showed more prominent expression in the fifth and ninth (int9L and int9R) intestinal rings. These expression profiles suggest that there are multiple paths to “dimness”, but only one path to “brightness” at the cellular level. Thus varying tissue specificity of *hsp-16.2* expression and/or quantitative differences could underlie some or all of the variation in longevity that distinguishes bright and dim worms in Rea et al. [1].

### Future Work

The current bottleneck in our studies is image acquisition. At present, each worm must be moved from culture medium to a separate slide and imaged separately, which is relatively time-consuming (about 30 minutes per worm) and prevents scaling up this approach to more than 15–20 worms per day per person. However, this may already be sufficient for some applications. On the other hand, processing these images needs 1-2 minutes per worm and is fully automatic, so it is already much faster (about 1440 images per day per single-core computer and easy to speed up by using more than one core and/or more than one computer)

One way to address this bottleneck would be to adopt chamber-slide-based microscopy coupled with automated imaging. This would allow increasing image resolution (by taking more images of each worm at higher magnification) as well as speeding up image acquisition by growing worms directly on chamber slides, anesthetizing them, and recording the whole population in one sweep. This would necessitate an extension of *pixelClassification* to recognize eggs, adult worms, head/tail/vulva anatomical features etc., and also extending *meshAB* to process more than two images. We have recently developed a stitching algorithm for multi-stained tissue samples in Heindl et al. [12] so the extension of *meshAB* would be quite straightforward. However, an extension of *pixelClassification* would still be quite challenging.

While we only tested the method on *Phsp-16.2::gfp*, it is likely to be applicable to other fluorescent reporters. To remove the dependency of worm segmentation on normalized GFP images, we are experimenting with using DIC as well as a combination of DIC and GFP images for worm segmentation and first results seem promising.

Even though our 2D intensity images give useful information on expression patterns and are straightforward to average and cluster, it would be useful to be able to map each intensity to one or a small set of underlying cells, thus generating cell-specific summaries of intensity patterns. However, such comprehensive 2D/3D cell identity/type maps do not yet exist (see next section).

Even if such 3D maps for the adult worm already existed, work would also need to be done on flexible non-linear worm deformation models to accurately map 2D images to such a 3D map. It should be noted that our present system manages to extract only one anatomically aligned intensity value per about 100 image pixels. We determined this maximum resolution by comparing two 2D intensity images from the same worm, see Section *Preparing 2D intensity images*, last paragraph. It seems clear that a linear worm straightening model is not able to align small anatomical features at a high resolution, especially as it is likely that both cell borders and features within the cell move in non-linear paths with respect to worm pose. This may also be responsible for a part of the errors reported in Long et al. [13]. The maximum resolution clearly needs to be increased to enable efficient use of 3D maps. It is likely that a limited form of 3D image reconstruction (such as 3D deconvolution from multiple images at different focus levels) will be necessary to obtain a sufficiently good alignment as the worm is obviously not two-dimensional.

### Related Work in 3D Atlases

Murray et al. [14] have developed a system to automatically trace cell lineage during embryogenesis of *C. elegans*. A dedicated laser scanning microscope is needed for each specimen. Ning et al. [15] have demonstrated a system to segment and track cells in *C. elegans* embryos just based on their DIC images, using convolutional neural networks for pixel classification and an energy-based clean-up function. They just need a dedicated light microscope. While promising, their approach has not been comprehensively tested and needs background knowledge about the structure of embryos. It also has been demonstrated only for the first few rounds in embryogenesis. Both approaches have not yet managed to track cells during the final round of embryogenesis. If they had, such data would give an accurate 3D map for the first larval state of *C. elegans* which still has only about half of the cells of a fully grown specimen. Both tracking methodologies rely on following cell division and thus cannot be used for already fully grown specimens.

Luengo Hendriks et al. [16] have developed a similar system for *Drosophila melanogaster*. Rather than using a dedicated laser scanning microscope as the previous approaches, they recorded different embryos with different staining at different timepoints. However, their method does not allow the identification or tracking of specific cells and is thus less powerful than the previous ones.

Long et al. [17] describe a graph-based algorithm to determine cell identities in a 3D confocal image of *C. elegans* based on their highly stereotyped arrangement and quote an average accuracy of 94.91%. Based on this work, Long et al. [13] have constructed a 3D map for the first larval stage of *C. elegans* (L1). However, they only report nuclear locations for a subset (63.97%) of all nuclei present at this developmental stage and do not give any cell border information. Also, the L1 stage has only about half of the cells of the adult specimen and therefore cannot be directly used to estimate cell type/positions in adult specimen.

## References

[1] Rea S, Wu D, Cypser JR, Vaupel JW, Johnson TE (2005). A stress-sensitive reporter predicts longevity in isogenic populations of Caenorhabditis elegans?. Nat Genet.

[2] Dupuy D, Bertin N, Hidalgo CA, Venkatesan K, Tu D (2007). Genome-scale analysis of in vivo spatiotemporal promoter activity in Caenorhabditis elegans.. Nature Biotechnology.

[3] Peng H, Long F, Liu X, Kim SK, Myers EW (2008). Straightening Caenorhabditis elegans images.. Bioinformatics.

[4] Guberman JM, Fay A, Dworkin J, Wingreen NS, Gitai Z (2008). PSICIC: noise and asymmetry in bacterial division revealed by computational image analysis at sub-pixel resolution.. PLoS Comput Biol.

[5] Burger W, Burge M (2008). Digital Image Processing: An Algorithmic Introduction Using Java,.

[6] le Cessie S, van Houwelingen JC (1992). Ridge Estimators in Logistic Regression.. Applied Statistics.

[7] Geng W (2004). Studying C. elegans phenotypes, PhD dissertation.

[8] Shimodaira H (2004). Approximately unbiased tests of regions using multistep-multiscale bootstrap resampling.. Annals of Statistics.

[9] Shimodaira H (2002). An approximately unbiased test of phylogenetic tree selection.. Systematic Biology.

[10] Suzuki R, Shimodaira H (2004). An application of multiscale bootstrap resampling to hierarchical clustering of microarray data: How accurate are these clusters?.

[11] R Development Core Team (2009). R: A Language and Environment for Statistical Computing.

[12] Heindl A, Ecker R, Steiner G, Bises G, Ellinger I (2009). Automated cell-detection technologies for science and diagnostics..

[13] Long F, Peng H, Liu X, Kim S, Myers G (2009). A 3D digital atlas of C. elegans and its application to single-cell analyses.. Nat Meth.

[14] Murray JI, Bao Z, Boyle TJ, Boeck ME, Mericle BL (2008). : Automated analysis of embryonic gene expression with cellular resolution in C. elegans.. Nat Methods.

[15] Ning F, Delhomme D, LeCun Y, Piano F, Bottou L (2005).

[16] Luengo Hendriks C, Keranen S, Fowlkes C, Simirenko L, Weber G (2006). Three-dimensional morphology and gene expression in the Drosophila blastoderm at cellular resolution I: data acquisition pipeline.. Genome Biology.

[17] Long F, Peng H, Liu X, Kim S, Myers G, Vingron M, Wong L (2008). Automatic Recognition of Cells (ARC) for 3D Images of C. elegans.. Proceedings of RECOMB 2008, LNBI 4955.

